# Orchestration of Genetic Alterations in *PSEN1* and *PSEN2* Genes in Development of Alzheimer's Disease through Computational Analysis

**DOI:** 10.1055/s-0043-1777849

**Published:** 2024-01-09

**Authors:** Asif Mir, Zainab Kamran, Wajid Iqbal

**Affiliations:** 1 Department of Biological Sciences, FBAS, International Islamic University, Islamabad, Pakistan

**Keywords:** dementia, *PSEN1* and *PSEN2*, mutational analysis, docking, phylogenetic history

## Abstract

Dementia is a syndrome that can cause a number of progressive illnesses that affect memory, thinking, and ability to perform everyday tasks. Alzheimer's disease (AD) is the most common cause of dementia and represents a major public health problem. AD is a progressive disease, where in early stages there is mild memory loss and in late-stage patient loses the ability to carry on a conversation. AD (for which there is no exact cause and cure known so far) is the sixth leading cause of deaths in the United States. Every 68 second someone develops AD. This study focuses on protein structure modeling of genes presenilin 1 and 2 ( *PSEN1* and *PSEN2* ) and their mutated forms (Asn141Tyr found in Chinese family, Gly34Ser identified in a Japanese patient, and Arg62Cys & Val214Leu identified in the Korean patients). It also involves wild and mutant type comparison, protein interaction studies, docking and phylogenetic history based on representative ortholog species and also sheds insight into the comparative evolutionary rates of coding sequence across various orthologs. This study gives a time and cost-effective analysis of genes ( *PSEN1* and *PSEN2* ) underlying AD and genetic alterations that drive development and causes of disease.

## Introduction

Alzheimer's disease (AD) is the neurodegenerative brain disease and the most common type of dementia. Dementia is a syndrome that can cause number of progressive illnesses that affects memory, thinking, and ability to perform everyday tasks. The disease is named after Alois Alzheimer who in 1906 identified changes in brain tissue of women who had died of mental illness. After she died, he examined his brain and found amyloid plaques and tangles in her brain. These plaques and tangles are the main causes of AD. The destruction of the neurons affects other part of the brain that affects the patient ability to do daily tasks. In the final stage of AD, patients are bed-bounded and require round-the-clock care and result in ultimately becoming fatal. AD is the sixth leading fatal disease in United States more than breast and prostate cancer. AD is of two types: early-onset and late-onset. Early-onset AD can be present in people aged between 30 and 60 years. The main reason of it is inherited mutation. A child whose mother or father carries a genetic mutation for early-onset AD has a 50% chance of inheriting that mutation. Late-onset AD can be present in people of aged more than 60 years. The reason for late-onset AD is still not understood. AD symptoms vary among individuals. The most common initial symptom is losing the ability to remember new information. AD patients may experience depression, social withdrawal, changes in sleeping habits, and mood swings. AD progresses slowly in three general stages: mild, moderate, and severe ( Table 1 ).

**Table 1:** Stage-wise progression of Alzheimer's disease

Mild stage	Moderate stage	Severe stage
Trouble in remembering names	Unable to recall their own address	Difficulty in eating
Difficulty in performing tasks	Confusion about where they are	Difficulty in communicating
Forgets that, one has just read	Risk of wandering and becoming lost	Lose awareness of recent experiences
Misplacing a valuable object	Difficulty in conversation	Require high levels of assistance with daily activities
Trouble with planning or organizing	Poor judgement	Become vulnerable to infections

AD patients can also experience depression, social withdrawal, changes in sleeping habits, and mood swings. According to APRC (Alzheimer's Pakistan Rawalpindi Chapter) in Pakistan, there is no population-based study on neurological diseases like AD. Due to a lack of research, it is very difficult to calculate an accurate number of people suffering from this AD. However, there are estimated 1 million Pakistanis who are living with AD and other forms of dementia according to APRC. To date there is no cure for AD. There are some medicines that slow down its progress especially in the early stages and others can help with mood changes and other behavior problems. Four genes involved in causing AD are PSEN1, PSEN2, APP, and APOE. Until now more than 200 mutations in gene encoding presenilin 1 ( *PSEN1* ) are described throughout the world and less than 40 mutations in gene encoding presenilin 2 ( *PSEN2* ) have been identified so far. Many *PSEN2* mutations were identified in European and in African populations. Only two were found in Korean populations and only four missense mutations in PSEN2 were found in Asia. *PSEN2* mutations are not only described in AD patients but also in patients with other disorders like frontotemporal dementia, breast cancer, and Parkinson's disease with dementia. Here, we have summarized the *PSEN2* missense mutations found in Asia.

Mostly imaging tests of the brain are in use to confirm that person is suffering from AD or not. Magnetic resonance imaging uses radio and magnets waves to make pictures of the brain and scans to show if someone has had a stroke or blood clots that might cause the symptoms. Positron emission tomography scan shows the plaques that build up in brain due to AD. To date there is no cure for AD. There are some medicines that slow down its progress especially in the early stages and others can help with mood changes and other behavior problems. The first drug that is approved by Food and Drud Administration for the treatment of Alzheimer's was tacrine (Cognex). It worked by slowing the breakdown of acetylcholine that helps nerve cells in the brain send messages to each other. In 2012, it was removed from market because it caused liver damage. Memantine (Namenda) is another drug that is used to slow down the Alzheimer's process by keeping brain cell from using too much of brain chemical called glutamate and protect against nerve damage. This drug has fewer side effects than other.

**Fig. 1:**
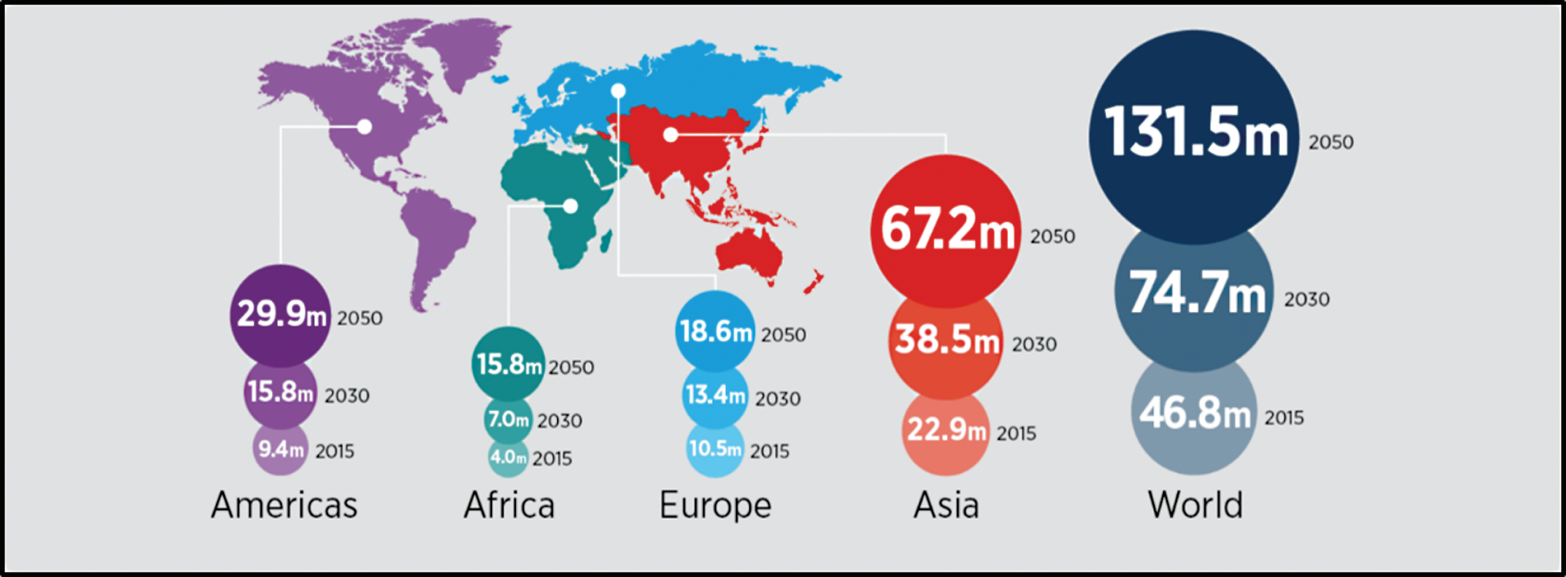
People living with dementia around the world.

There are estimated 1 million Pakistanis who are living with AD and other forms of dementia according to APRC (Alzheimer's Pakistan Rawalpindi Chapter). The number of Americans suffering from AD is growing fast. An estimated 5.4 million Americans are suffering from AD. People aged 70 years who have AD have 61% chance to die before the age of 80 years. A worldwide estimation of dementia has been shown in Fig. 1 .

AD causes estimated 60 to 70% cases of dementia. It is a neurodegenerative disease that acts chronically getting worse with the passage of time 1 (World Health Organization, 2012). Short-term memory loss is the most common early event seen among the patients, which results in difficulty remembering the recent events. 1 With the development of disease, several events can occur that include loss of motivation, mood swings, unable to maintain self-care, and other behavioral issues including language problems. 1 (World Health Organization, 2012). As the condition worsens, the patient withdraws himself/herself from society as well as family. 1 Before the death of patients, body parts stop functioning gradually overtime (National Institute on Aging, 2011). It has been observed that life expectancy of AD can vary from patients to patient, but on average a period of 3 to 9 years between diagnosis and complete progression of disease is observed (Querfurth & LaFerla, 2010). 2

Research over the years has shown that causes of AD are not well understood. 1 It is thought that 70% of the risk associated with the disease is genetic that involves several genes. 3 Risk factors other than genetics associated with this disease include a history of hypertension, head injuries, or depression. 1 The progression of disease is associated with tangles and plaques in the brain. 3 For the purpose of diagnosis, a history of illness, blood tests as well as cognitive testing with medical imaging can be used to rule out the other possible causes (National Institute for Health and Care Excellence (NICE), 2016). At initial stage, symptoms can be mistaken for normal aging. 1 For a proper and timely diagnosis, an examination of brain tissue can be helpful. However, it has been observed that physical as well as mental exercise and avoiding obesity can decrease the risk of AD. 4 However, research has shown that there are no effective medications or other such materials that decrease the risk of disease development (National Institute on Aging, 2006).

## Materials and Methods

**Fig. 2:**
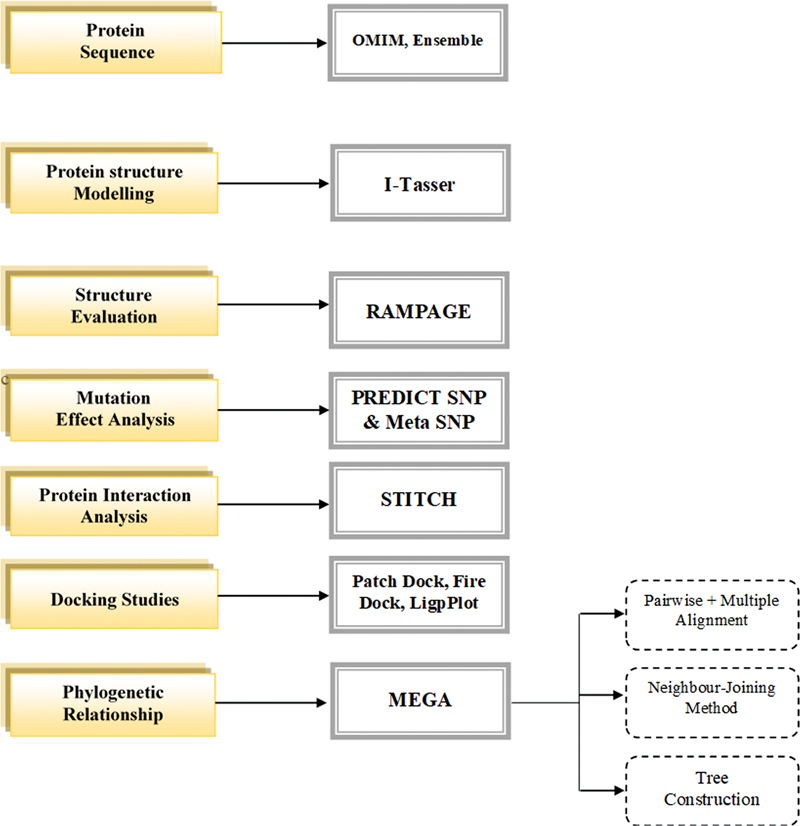
Flowchart of methodology used during study.

A graphical representation of work done in current study has been shown in Fig. 2 . Three-dimensional structures of *PSEN1* and *PSEN2* (wild and mutated forms) were built using I-Tasser. We used RAMPAGE server 5 for the evaluation of three-dimensional models (wild + Mutated) of protein. Meta-SNP 6 and PREDICT SNP 4 were utilized to estimate the effect of mutation on stability of protein and to determine whether the mutation has an impact on normal function of protein or not. The evolutionary history was inferred using the Neighbor-Joining method 7 using software MEGA. 8 Sequences of query gene PSEN1, PSEN2, and of all orthologs were collected through ensemble database. Sequence similarity of ortholog species with human gene sequence were analyzed through alignment using basic local alignment search tool) Johnson et al., 2008 4 9 against the protein database to choose closest putative orthologous protein sequences. After sequence acquisition, pair-wise and multiple alignment was performed using ClustalW algorithm. The algorithm calculates similarity percentage between sequences and generates an alignment file that is further used as input file during tree reconstruction. The bootstrap consensus tree inferred from 1000 replicates is taken to represent the evolutionary history of the taxa analyzed. The branches were collapsed which corresponding to partitions reproduced in less than 50% bootstrap replicates. The percentage of replicate trees in which the associated taxa clustered together in the bootstrap test (1000 replicates) us shown next to the branches. P-distance method (chosen as a substitution model) was used to calculate evolutionary distances and these distances are in the units of the number of amino acid differences per site. The current analysis of *PSEN1* and *PSEN2* genes involved 16 amino acid sequences, that is, one target sequence of human gene and 15 ortholog species from different categories. 10

## Results

**Fig. 3:**
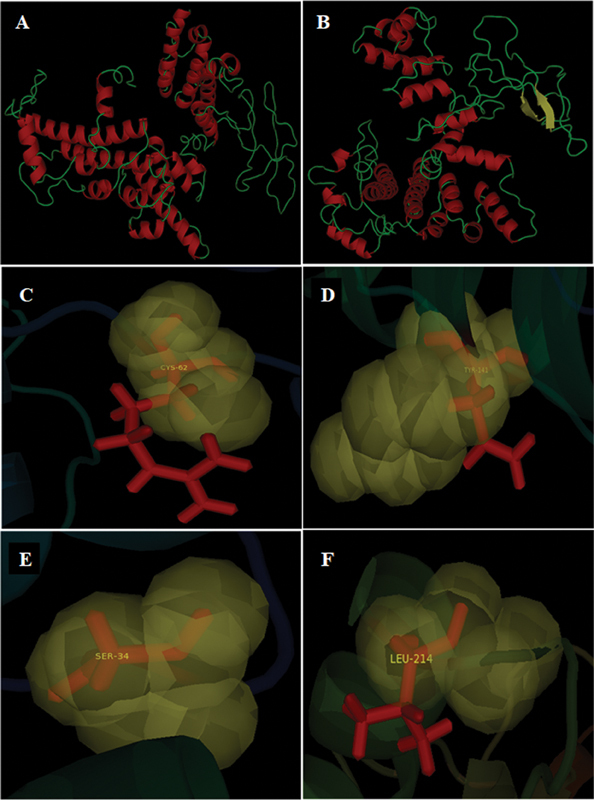
**Three-dimensional** structures: ( **A** ) *PSEN1* and ( **B** ) *PSEN2* . Structures have been shown in cartoon displaying style via visualizer PyMol. Alpha helices as Coils (shown in Red Color), coils (shown in green color) and Beeta sheets as flat ribbons (shown in yellow color). Super imposed models to highlight mutations ( **C** ) Arg62Cys, ( **D** ) Asn141Tyr, ( **E** ) Gly34Ser, & ( **F** ) Val214Leu. Wild-type amino acids have been shown in red sticks and substituted amino acid shown in yellow sphere display style with label on it.

The three-dimensional models of *PSEN1* and *PSEN2* are modeled by using I-Tasser ( Fig. 3A and B ). Mutations, Arg62Cys, Asn141Tyr, Gly34Ser, and Val214Leu, have been highlighted by superimposing the normal and mutated structures of PSEN2 (as shown in Fig. 3C–F ). Evaluation of structures ( Table 2 ) revealed that these are reliable as maximum number/percentage of amino acids lie in the favored region that is a sterically allowed area in Ramachandran plot. This analysis not only determined reliability of structures but also showed how normal structure changes upon mutation. 11 12 13

**Table 2:** Evaluation of structures through RAMPAGE

Type	Structures	Validation using RAMPAGE		
		Favored region	Allowed region	Outlier region
Normal	PSEN1	83.0%	11.3%	5.7%
	PSEN2	87.3%	5.8%	6.9%
Mutated PSEN2	Arg62Cys	87.6%	5.5%	6.9%
	Asn141Tyr	87.3%	6.3%	6.3%
	Gly34Ser	87.6%	5.5%	6.9%
	Val214Leu	87.1%	6.1%	6.9%

### Protein Stability Analysis

**Fig. 4:**
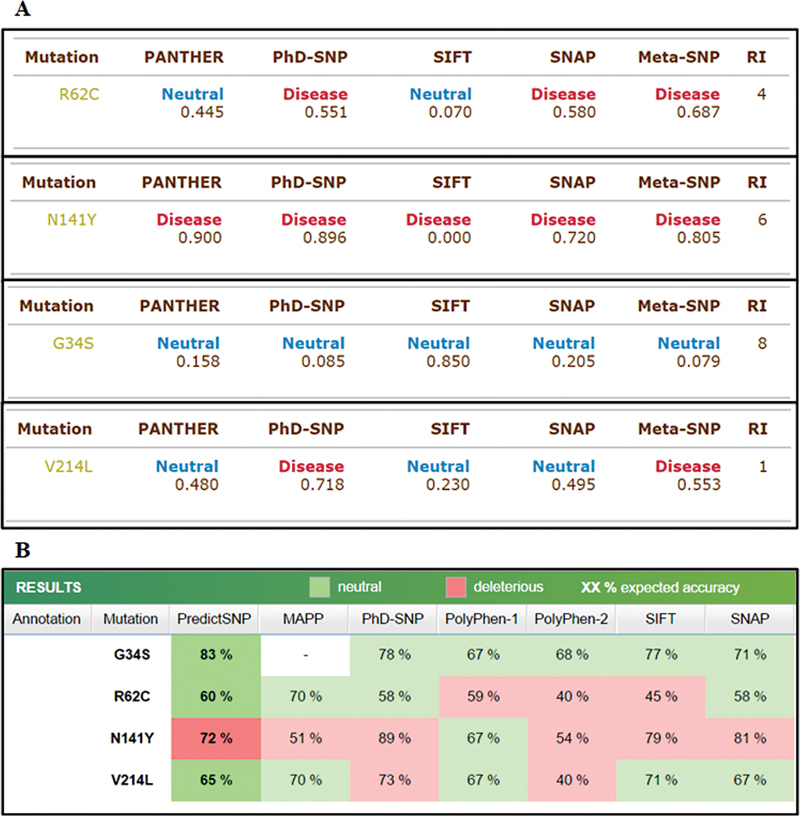
FGFR3 mutation (p.G380R) effect prediction: ( **A** ) PREDICT SNP results and ( **B** ) Meta-SNP result. FGFR, fibroblast growth factor receptor.

PREDICT SNP and Meta-SNP were also utilized to analyze effect of mutation on protein, results of which are shown in Fig. 4 . PREDICT SNP combines six best performing prediction methods to provide more accurate and robust alternative to the predictions delivered by individual integrated tools. Mutations with deleterious effect correspond to natural variants with known clinical manifestation. This is in many cases accompanied by decreased stability of the protein. On the other hand, mutations predicted as neutral mostly correspond to natural variants without known negative effects. Meta-SNP also hires with it different predictors (PATHER, PhD-SNP, SIFT, and SNAP) to calculate mutation impact on normal protein. Value reported under each prediction ( Fig. 4 ) is given below. Maximum number of predictors show it as disease causing which validate our result.

- PANTHER: Between 0 and 1 (if >0.5, mutation is predicted disease)
- PhD-SNP: Between 0 and 1 (if >0.5, mutation is predicted disease)
- SIFT: Positive value (if >0.05, mutation is predicted neutral)
- SNAP: Output normalized between 0 and 1 (if >0.5, mutation is predicted disease)
- Meta-SNP: Between 0 and 1 (if >0.5, mutation is predicted disease)

### Estimation of Evolution

**Fig. 5:**
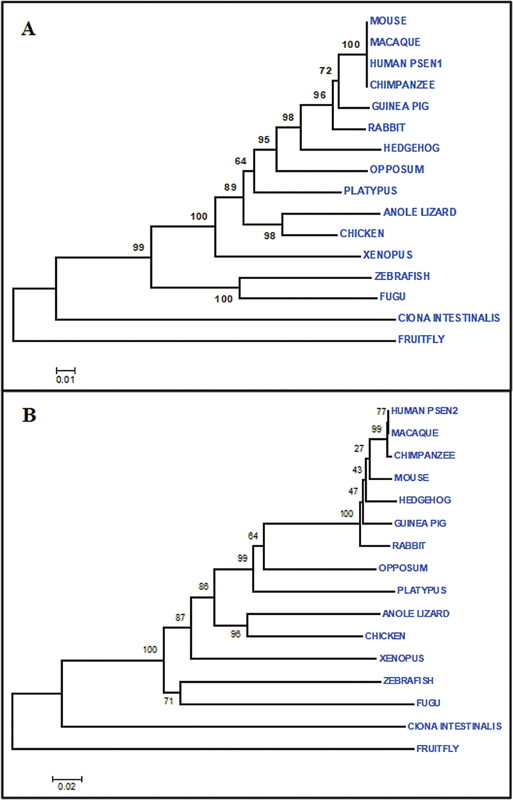
MEGA results: Evolutionary relationships through Neighbor Joining Method: ( **A** ) *PSEN1* , ( **B** ) *PSEN2* .

This study showed the phylogenetic history of *PSEN1* and *PSEN2* based on representative ortholog species and also shed insight into the comparative evolutionary rates of coding sequence across various orthologs. Neighbor joining trees for *PSEN1* and *PSEN2* are shown in Fig. 5 and both target genes show close relatedness with chimpanzee, macaque, and mouse. According to *PSEN1* tree, human is making cluster with chimpanzee, mouse, and macaque with bootstrap value of 100. Other clusters with highest bootstrap value i.e., 100 include cluster of Fugu and zebrafish and *Xenopus* with anole lizard/chicken/platypus/opossum/rabbit/Hedgehog/guinea pig / chimpanzee / human/ macaque/ mouse. High bootstrap values of the clusters correspond to highest reliability. Tree is reconciling species divergence time. Fruit fly and Ciona intestinalis are as outgroup in this tree. Evolutionary time for the tree is 0.01. Human is making cluster with macaque with bootstrap value of 77 in *PSEN2* gene tree ( Fig. 5 ). Clusters with highest bootstrap value i.e.100 include cluster of Rabbit with Guinea Pig/ Hedgehog/ Mouse/ Chimpanzee/ Macaque/ Human and Fugu/Zebrafish with *Xenopus* with Chicken/Anoli Lizard/ Platypus/ Oppossum/ Rabbit/ Guinea Pig/ Hedgehog/ Mouse/ Chimpanzee/ Macaque/ Human. High bootstrap values of the clusters correspond to highest reliability. This gene tree is also reconciling species divergence time. Fruit fly and Ciona intestinalis are as outgroup in this tree. Evolutionary time for the tree is 0.02.

### Interaction Studies

**Fig. 6:**
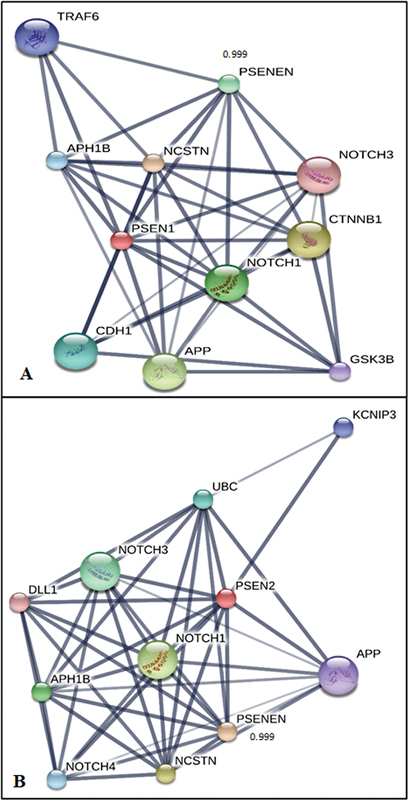
Stitch results for protein interaction network study of *PSEN1* and *PSEN2* .

**Fig. 7:**
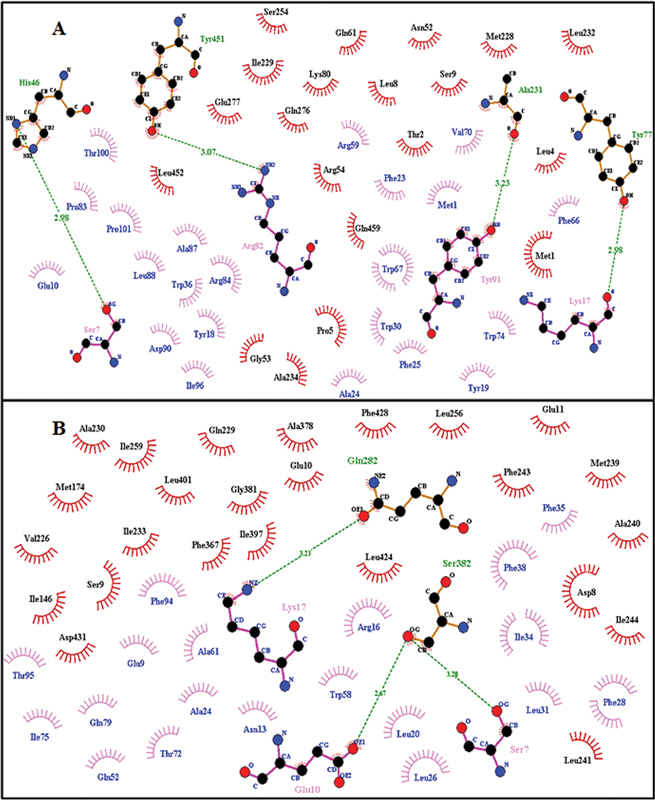
Two-dimensional Ligplot representation of Docking Interaction: ( **A** ) *PSEN1* with PSENEN, *(* **B** ) *PSEN2* with PSENEN.

**Fig. 8:**
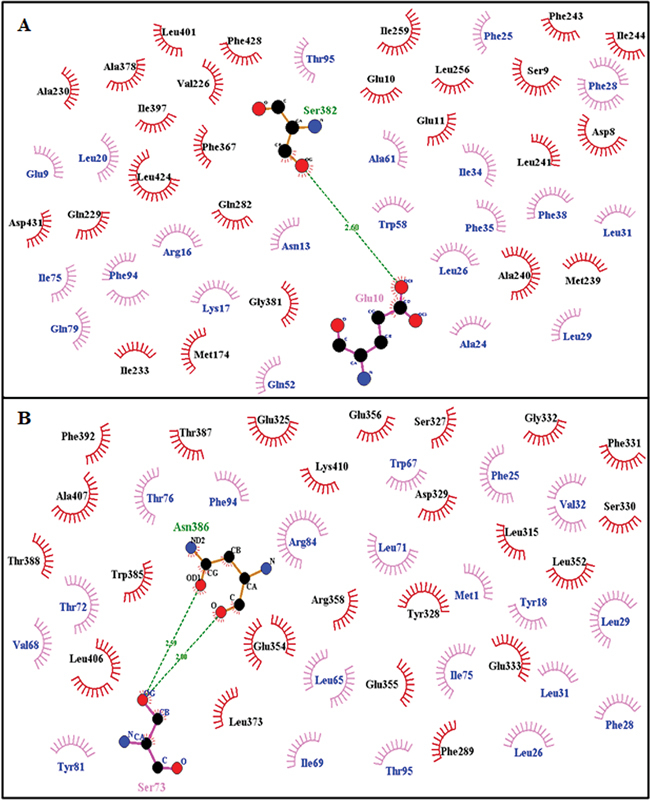
Two-dimensional Ligplot representation of Docking Interaction: ( **A** ) Arg62Cys with PSENEN, ( **B** ) Asn141Tyr with PSENEN.

**Fig. 9:**
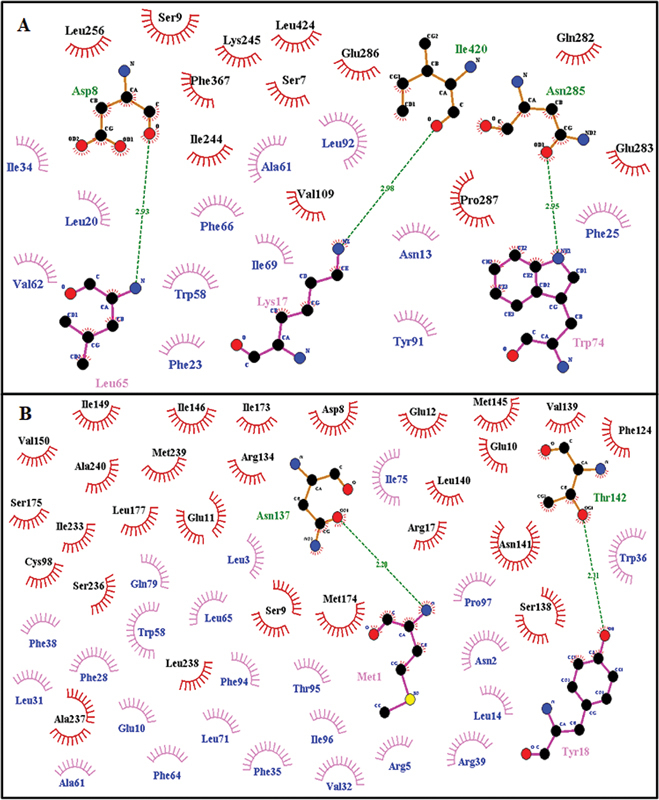
Two-dimensional Ligplot representation of Docking Interaction: **A** ) Gly34Ser with PSENEN, ( **B** ) Val214Leu with PSENEN.

PSENEN presenilin enhancer 2 homolog (ID: ENSP00000222266) with interaction score of 0.999 has been found to be closest relative for both genes ( Fig. 6 ) as per stitch results. It consists of 101 amino acids. It is an essential subunit of the gamma–secretase complex, an endoprotease complex that catalyzes the intramembrane cleavage of integral membrane proteins such as Notch receptors and APP (β-amyloid precursor protein). It probably represents the last step of maturation of gamma-secretase, facilitating endoproteolysis of presenilin and conferring gamma-secretase activity. Dimplot results for the docking interactions of *PSEN1* and *PSEN2* with ligand (PSENEN) are shown in Fig. 7A and B . Interaction of mutated protein (Arg62Cys) with PSENEN as shown in Fig. 8A and B shows Asn141Tyr with PSENEN. Fig. 9A and B shows interaction of Gly34Ser with PSENEN and Val214Leu with PSENEN. Residues involved in hydrogen bonding and hydrophobic interactions for all types of interactions have been summarized in Table 3 . Differences in residues and their positions involved in both types of docking interactions clearly revealed that how active site deviation occurred and effected protein interaction as a result of mutation.

**Table 3:** Docking interactions summary: residues involved in hydrogen bonding and hydrophobic interactions

Receptor-ligand	Hydrogen bond interactions		Hydrophobic interactions		Figure
	Ligand residues	Receptor residues	Ligand residues	Receptor residues	
PSEN1 **-** PSENEN	Ser7, Lys17, Arg82, Tyr91	His46, Tyr77, Ala231, Tyr451	Met1, Glu10, Tyr18, Tyr19, Phe23, Ala24, Phe25, Trp30, Trp36, Arg59, Phe66, Trp67, Val70, Trp74, Pro83, Arg84, Ala87, Leu88, Asp90, Ile96, Thr100, Pro101	Met1, Thr2, Leu4, Pro5, Leu8, Ser9, Asn52, Gly53, Arg54, Gln61, Lys80, Met228, Ile229, Leu232, Ala234, Ser254, Gln276, Glu277, Leu452, Gln459	
PSEN2 - PSENEN	Ser7, Glu10, Lys17	Gln282, Ser382	Glu9, Asn13, Arg16, Leu20, Ala24, Leu26, Phe28, Leu31, Ile34, Phe35, Phe38, Gln52, Trp58, Ala61, Thr72, Ile75, Gln79, Phe94, Thr95	Asp8, Ser9, Glu10, Glu11, Ile146, Met174, Val226, Gln229, Ala230, Ile233, Met239, Ala240, Leu241, Phe243, Ile244, Leu256, Ile259, Phe367, Ala378, Gly381, Ile397, Leu401, Leu424, Phe428, Asp431	
PSEN2- Arg62Cys	Glu10	Ser382	Glu9, Asn13, Arg16, Leu20, Ala24, Phe25, Leu26, Phe28, Leu29, Leu31, Ile34, Phe35, Phe38, Gln52, Trp58, Ala61, Ile75, Gln79, Phe94, Thr95	Asp8, Ser9, Glu10, Glu11, Met174, Val226, Gln229, Ala230, Ile233, Met239, Ala240, Leu241, Phe243, Ile244, Leu256, Ile259, Gln282, Phe367, Ala378, Gly381, Ile397, Leu401, Leu424, Phe428, Asp431,	
PSEN2- Asn141Tyr	Ser73	Asn386	Met1, Tyr18, Phe25, Leu26, Phe28, Leu29, Leu31, Val32, Leu65, Trp67, Val68, Ile69, Leu71, Thr72, Ile75, Thr76, Tyr81, Arg84, Phe94, Thr95,	Phe289, Leu315, Glu325, Ser327, Tyr328, Asp329, Ser330, Phe331, Gly332, Glu333, Leu352, Glu354, Glu355, Glu356, Arg358, Leu373, Trp385, Thr387, Thr388, Phe392, Leu406, Ala407, Lys410	
PSEN2- Gly34Ser	Lys17, Trp74	Asn285, Ile420	Asn13, Leu20, Phe23, Phe25, Ile34, Trp58, Ala61, Val62, Phe66, Ile69, Tyr91, Leu92	Ser7, Ser9, Val109, Ile244, Lys245, Leu256, Gln282, Glu283, Glu286, Pro287, Phe367, Leu424	
PSEN2- Val214Leu	Met1, Tyr18	Asn137, Thr142	Asn2, Leu3, Arg5, Glu10, Leu14, Phe28, Leu31, Val32, Phe35, Trp36, Phe38, Arg39, Trp58, Ala61, Phe64, Leu65, Leu71, Ile75, Gln79, Phe94, Thr95, Ile96, Pro97	Asp8, Ser9, Glu10, Glu11, Glu12, Arg17, Cys98, Phe124, Arg134, Ser138, Val139, Leu140, Asn141, Met145, Ile146, Ile149, Val150, Ile173, Met174, Ser175, Leu177, Ile233, Ser236, Ala237, Leu238, Met239, Ala240,	

Receptor Residues Involved in Hydrophobic Interactions are represented by brick red spoked arcs (  ), Hydrogen Bonding shown by Green dotted lines (  ). Receptor residues involved in H-bonding are shown in olive green color. Ligand residues involved in H-bonding are shown in blue color.

## Conclusion

The rich amount of genomic sequence data available enables computational analysis of target genes of interest and to provide important and useful insight into their normal and mutational pathway, their link with specific phenotypic characteristic as well as association with human disease. AD is a complicated neurodegenerative disorder whose causes and effects are yet to be understood. This study gives a time and cost-effective analysis of genes ( *PSEN1* and *PSEN2* ) underlying AD and appears to help orchestrate the genetic alterations that drive development and causes of disease. Structures for normal and mutated forms of proteins have been modeled, compared, and analyzed with respect to impact. Mutational analysis provided insight into how changes in normal protein lead to disease state. Evolutionary relationship for both genes was determined by reconstruction of phylogenetics trees. Classification with respect to various orthologs explored the ancestor and descendent relationship.
